# Improved MRI methods to quantify retinal and choroidal blood flow applied to a model of glaucoma

**DOI:** 10.3389/fopht.2024.1385495

**Published:** 2024-05-13

**Authors:** Zhao Jiang, Diane Chernoff, Andre Galenchik-Chan, David Tomorri, Robert A. Honkanen, Timothy Q. Duong, Eric R. Muir

**Affiliations:** ^1^ Department of Radiology, Stony Brook University, Stony Brook, NY, United States; ^2^ Renaissance School of Medicine at Stony Brook University, Stony Brook, NY, United States; ^3^ School of Health Professions, Stony Brook University, Stony Brook, NY, United States; ^4^ Department of Ophthalmology, Stony Brook University, Stony Brook, NY, United States; ^5^ Department of Radiology, Albert Einstein College of Medicine, Bronx, NY, United States; ^6^ Department of Radiology, The University of North Carolina at Chapel Hill, Chapel Hill, NC, United States

**Keywords:** MRI, blood flow, glaucoma, imaging, retina, choroid, mouse

## Abstract

**Purpose:**

Blood flow (BF) of the retinal and choroidal vasculatures can be quantitatively imaged using MRI. This study sought to improve methods of data acquisition and analysis for MRI of layer-specific retinal and choroidal BF and then applied this approach to detect reduced ocular BF in a well-established mouse model of glaucoma from both eyes.

**Methods:**

Quantitative BF magnetic resonance imaging (MRI) was performed on glaucomatous DBA/2J and normal C57BL/6J mice. Arterial spin labeling MRI was applied to image retinal and choroidal BF using custom-made dual eye coils that could image both eyes during the same scan. Statistics using data from a single eye or two eyes were compared. BF values were calculated using two approaches. The BF rate per quantity of tissue was calculated as commonly done, and the peak BF values of the retinal and choroidal vasculatures were taken. Additionally, the BF rate per retinal surface area was calculated using a new analysis approach to attempt to reduce partial volume and variability by integrating BF over the retinal and choroidal depths.

**Results:**

Ocular BF of both eyes could be imaged using the dual coil setup without effecting scan time. Intraocular pressure was significantly elevated in DBA/2J mice compared to C57BL/6J mice (P<0.01). Both retinal and choroidal BF were significantly decreased in DBA/2J mice in comparison to the age-matched normal C57BL/6J mice across all measurements (*P* < 0.01). From simulations, the values from the integrated BF analysis method had less partial volume effect, and from *in vivo* scans, this analysis approach also improved power.

**Conclusion:**

The dual eye coil setup allows bilateral eye data acquisition, increasing the amount of data acquired without increasing acquisition times *in vivo*. The reduced ocular BF found using the improved acquisition and analysis approaches replicated the results of previous studies on DBA/2J mice. The ocular hypertensive stress-induced BF reduction found within these mice may represent changes associated with glaucomatous progression.

## 1 Introduction

There are multiple imaging modalities to quantify blood flow (BF) and ocular perfusion, including laser Doppler velocimetry, Doppler ocular coherence tomography, and laser speckle imaging. However, most of these imaging modalities are depth limited, as most blood signals are associated with the retinal surface, such that choroidal blood flow cannot be analyzed due to its position behind the retinal pigment epithelium. Similar optical techniques have been utilized in rodents to assess BF but are mostly limited to the retinal vasculature (1, 2). BF of the retina and choroid in animal models has also been studied using destructive techniques, such as microspheres, which have yet to be applied to the mouse eye due to quantification issues with such small tissue samples (3, 4). Alternatively, MRI can provide non-invasive quantification of volumetric BF of the retina and choroid without depth limitation. However, there have been some limitations with prior applications of the MRI approach, including using hardware that could only assess one eye at a time and complications with BF calculations from the thin retina.

Previous studies have demonstrated the capability of MRI to detect ocular BF reduction in several rodent models of retinal disease and pathology, such as glaucoma and diabetes (5, 6). For example, Lavery et al. utilized MRI to investigate BF in the retinal and choroidal circulations in the established DBA/2J mouse model of glaucoma, which spontaneously develops progressive ocular hypertension and optic neuropathy with age (5). Compared to healthy C57BL/6J mice, ocular BF was found to be significantly lower (5), and intraocular pressure (IOP) was found to be significantly increased with age (7, 8). DBA/2J mice model a slower progressive glaucoma, more similar to the human disease. As MRI provides quantitative BF in absolute units and is non-invasive, it could provide utility to track slow longitudinal changes over months or years. Similar findings are consistent across both retinal and choroidal BF and have been supported in animal models and humans, suggesting a link between BF and glaucoma (9–11).

Glaucoma, a leading cause of irreversible blindness worldwide (12), is characterized by optic nerve degeneration and retinal ganglion cell death (13). The exact underlying mechanisms leading to ganglion cell loss in glaucoma patients are still not fully understood. One proposed cause is impaired ocular perfusion that may be caused by elevated IOP and systemic vascular abnormalities such as hypotension and vasospasm (9). Further, the endothelin-1 model of chronic optic nerve ischemia has shown that chronic BF deficit can cause optic neuropathy consistent with glaucoma (14). The decreased ocular perfusion may contribute to optic nerve degeneration and ganglion cell loss, leading to progressive vision loss (9). The primary site of neuropathy in glaucoma patients is the optic nerve head, which may have decreased perfusion (15), potentially depriving it from needed oxygen and nutrient content to meet its metabolic demands (15, 16). Impaired ocular hemodynamics have been reported in human glaucoma patients, as well as reduced microvascular density of the retina and optic disc (9, 11, 17).

The specific impairments of the retinal and choroidal vasculature in diseases such as glaucoma may depend in part on their different regulatory control. The retinal vasculature supplies the inner retina including the ganglion cell layer, with capillaries extending into the inner nuclear and inner plexiform layers. The choroidal vasculature is located between the retinal pigment epithelium and sclera and supplies the majority of the oxygen for the photoreceptors (18). The retinal vessels demonstrate BF regulation and neurovascular coupling to sustain retinal metabolic activity (19), with the vessels dilating in response to visual stimulations (20). It is unclear if the choroid is regulated by the local metabolic status of the tissue as the vessels are separated from the outer retina, however both retinal and choroidal vessels respond to hypercapnia (21, 22). The retinal vessels do not contain neural innervation, while the choroid is innervated directly by the autonomic nervous system (18, 19). Both vasculatures have been found to have myogenic autoregulation to maintain BF over a range of ocular perfusion pressure (23, 24), although a lack of autoregulation in the choroid has also been reported (21, 25), showing the need for further research (18, 19). The exact vascular pathophysiology in glaucoma and the underlying molecular mechanisms in the retina and choroid remain uncertain, which would need to be studied in disease models. Imaging technologies that can non-invasively quantify absolute BF of the retina and choroid separately could help to better understand the vascular pathophysiology of ocular diseases such as glaucoma.

Previous studies using MRI to evaluate ocular BF in rodent models have been limited to a single eye due to using a singular coil and using calculations that may be susceptible to partial volume effects, as in several reports showing progressive decline of retinal and choroidal BF in DBA/2J mice (5, 26). In this study, we strive to improve methods for receiving and analyzing BF MRI data to be applied to further support the hypothesis that ocular BF is reduced in DBA/2J mice. We introduce a novel setup utilizing dual imaging coils, enabling the simultaneous imaging of both eyes. This method streamlines data collection and essentially halves the scan time compared to repositioning the subject and coil for sequential eye scanning. This method could potentially reduce the necessary sample size due to increased statistical power, while providing significant results that are consistent with established methods of BF measurement.

Furthermore, a new method for quantifying retinal BF was used, in addition to the common quantification in units of flow per quantity of tissue (mL/mL/min). This method calculates flow per unit of tissue surface area (µL/mm²/min), units which have been utilized in animal studies using microspheres and autoradiography (27, 28). As the retina and choroid are bordered by avascular regions, partial volume effects should be reduced by integrating BF measurements over the depth of each vascular layer. The goal of the methods described herein is to assess the efficacy of the dual coil BF measurement technique (as opposed to singe coil) and the new method of BF quantification. We hypothesize that decreased bilateral ocular BF will be detected with improved statistical power in the DBA/2J mice compared to age-matched C57BL/6J mice.

## 2 Materials and methods

### 2.1 Animal preparation

The protocol was approved by the Institutional Animal Care and Use Committee at Stony Brook University in accordance with the Guide for the Care and Use of Laboratory Animals. Twelve DBA/2J and twelve C57BL/6J male mice (Jackson Laboratories, Bar Harbor, ME) between 10.5 to 12.5 months of age were used. The DBA/2J mouse model is a well-established genetic model of glaucoma. The mice have spontaneous mutations in two genes, *Tyrp1* and *Gpnmb*, which cause iris atrophy and pigment dispersion, leading to a slow, progressive elevation of IOP (7, 8, 29, 30). The progression of IOP elevation, optic nerve axonal damage, retinal ganglion cell loss, and functional deficits has been well-characterized in the model (7, 8, 31–34). The earliest changes begin around 4 to 5 months of age and become severe by 10 months of age and older (7, 31).

Animals were housed in institutional animal facilities in typical mouse cages under a reversed 12hr/12hr light/dark cycle (light 7 pm to 7 am) and received a standard rodent diet. Studies were conducted during the middle of the dark period between 9am to 4pm, with several mice being imaged during this period on a given day. The average time of measurements were about 12:45pm ± 2.2hr and 12:55pm ± 2.1hr (mean ± standard deviation) for DBA/2J and C57BL/6J mice, respectively, and the timing was not significantly different between groups (P=0.84 from t-test). IOP was measured by a rebound tonometer for rodents (Tonolab, iCare, Helsinki, Finland), with the device giving the average of six measurements taken per eye. IOP was measured directly after induction of anesthesia and prior to moving the animal into the MRI scanner, with about 30 to 45 min between IOP and BF measurements. For MR imaging, the animals were anesthetized with 1.6% isoflurane in room air and allowed to breathe spontaneously. To maintain a target respiratory rate of 80 to 120 breaths/minute, adjustments were made to the anesthesia level as needed. The head was immobilized with ear and tooth bars to reduce movement during imaging. Additionally, temperature was monitored, and animals were kept warm throughout the experiment with a water pad that circulated warm water to maintain a temperature of 37°C.

The data were discarded from analysis for the left eye of one DBA/2J due to a deformed eye and the right eye from another DBA/2J due to severe motion artifacts.

### 2.2 MRI measurements

**Figure 1:**
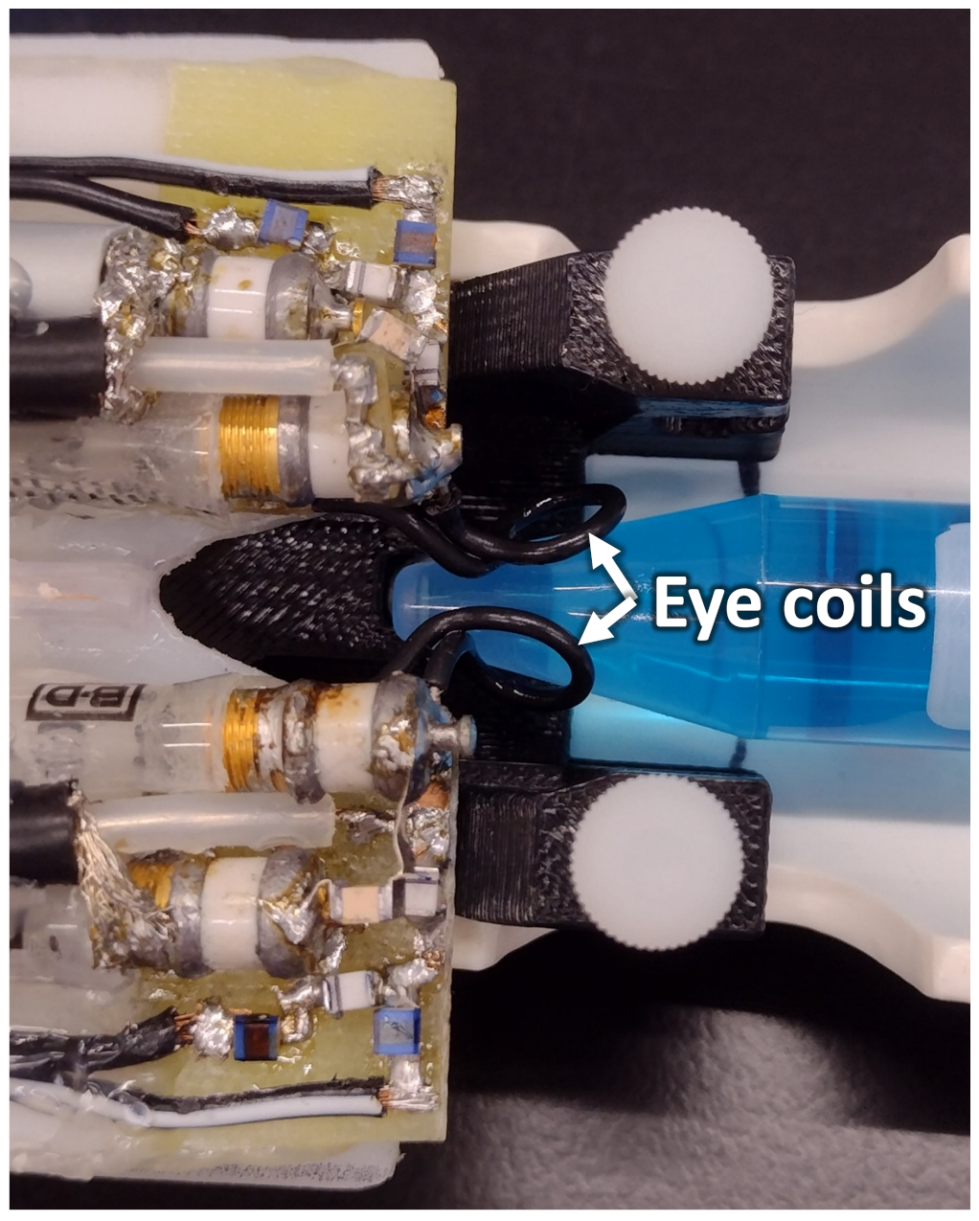
The setup of the dual eye coils on a phantom is shown. The loops of the two surface coils are indicated by the arrows. The two coils are connected by a tee, and active decoupling is used to prevent interference between the two transmit-receive imaging coils.

MRI was performed on a 7 Tesla scanner (Biospec; Bruker, Billerica, MA) with 600 mT/m gradients. Custom-made transmit/receive eye coils (6 mm diameter) for the left and right eye were used for imaging (**Figure 1**). Both coils had active detuning circuits, which were used to detune one coil while the other was transmitting and receiving. When a coil is detuned, it is switched far off from the resonance frequency which essentially eliminates interference between coils. The cables of the coils were cut to 1.5 wavelengths at 300 MHz and connected by tee to a single RF transmit/receive channel. Each coil was tuned and matched separately, while the other coil was detuned. For BF imaging, a circular heart coil (8mm diameter) was used for arterial spin labeling (ASL), connected to a second RF transmit channel. The dual eye coils were actively detuned during labeling.

The signal to noise ratio (SNR) and transmit efficiency of the dual coil setup was compared to single coils on a phantom. Scans were acquired in three configurations: with a single coil, with both coils placed but only one connected to the RF channel, and with both coils placed and both connected to the RF channel. The transmit power to achieve a 90° flip angle at the surface of the phantom was manually optimized for each coil using a RARE scan. The power for the 90° pulse was recorded for each configuration to assess potential transmission losses with both coils. SNR was measured from both FLASH and RARE scans with geometrical parameters of field of view (FOV) = 16x16 mm^2^, matrix = 160x160, and a single slice with thickness of 1 mm. Other imaging parameters for FLASH were a repetition time (TR) = 125 ms, echo time (TE) = 4 ms, and flip angle = 32°. Parameters for RARE were TR = 2000 ms, effective TE = 64 ms, and echo train length of 16.

Depth-resolved quantitative BF MRI was acquired with a gradient-echo, echo-planar imaging sequence with an FOV = 6x6 mm^2^ and 144x144 matrix, giving 42x42 µm^2^ resolution (35). The BF sequence used two non-contiguous 500 µm coronal slices, one through each eye. The slices were positioned at the optic nerve head and angled to be perpendicular to the retina. Other parameters were TR = 3000 ms, TE = 9 ms, partial Fourier of 2/3, 4 shots, and readout bandwidth = 170 kHz. Continuous ASL used a 2542 ms labeling pulse in the presence of a 20 mT/m gradient with post-labeling delay times for the left and right eyes of 325 and 381 ms, respectively. BF values were calculated from 75 repetitions acquired over 30 min and averaged offline. An image with equilibrium magnetization, M_0_, was acquired for BF calculation similarly but with a long TR = 10 sec and 2 repetitions.

Data analysis was performed with custom software (in Matlab, MathWorks Inc, Natick, MA). Motion correction was first performed using Statistical Parametric Mapping software (SPM12, Wellcome Centre for Neuroimaging, University College London, UK) (36). A semi-automated process was used to linearize the retina, perform further motion correction for more subtle eye motion, and conduct an automated profile analysis (37, 38). Profiles across the retinal depth were obtained from images by projecting lines perpendicular to the retina with profiles obtained at 6x spatial interpolation. The BF (mL/mL/min) was calculated from the difference between labeled and non-labeled images as previously reported (39–41) as

**Figure 2:**
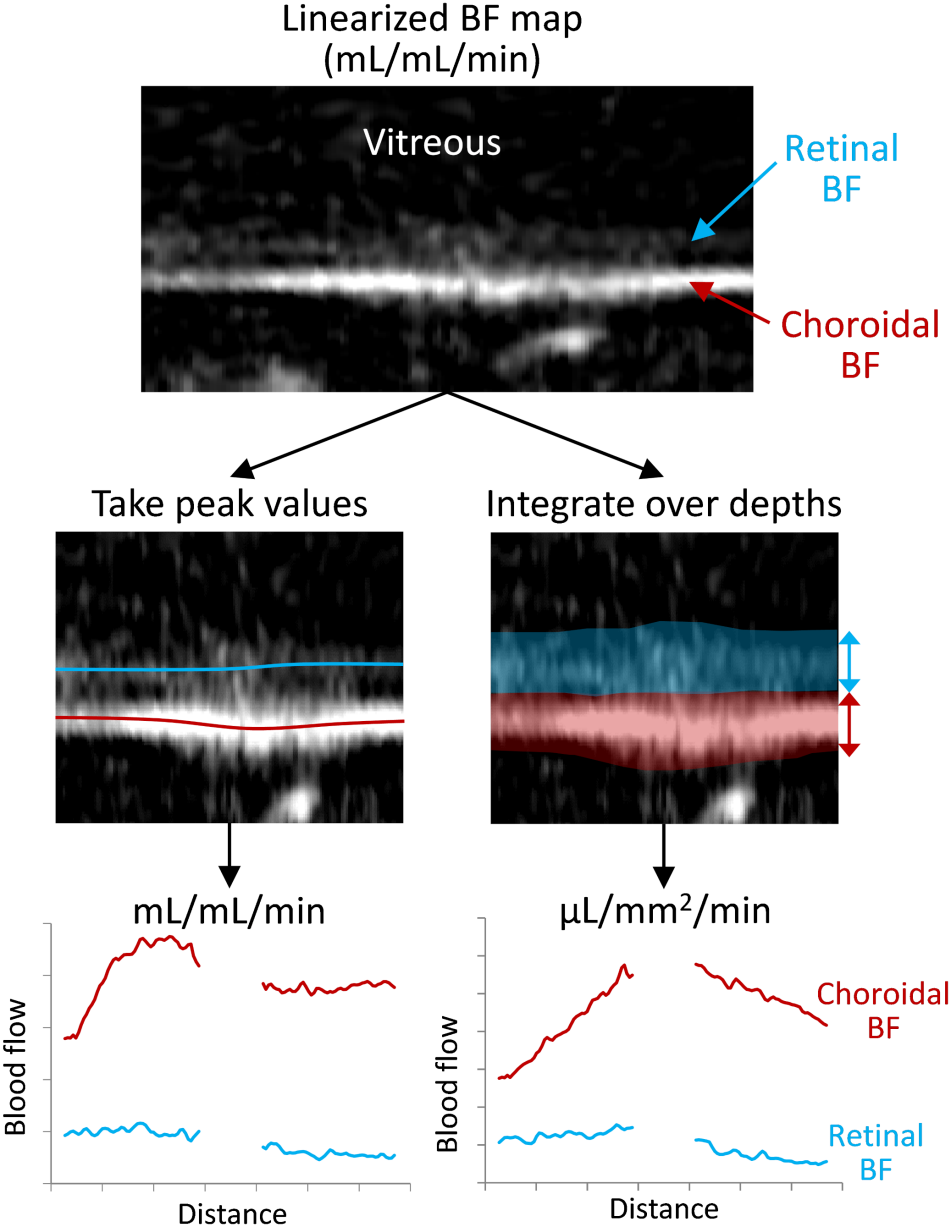
Depiction of the processing for the two analysis methods for taking the peak or the integrated blood flow values. The retina is virtually flattened, and the retinal/choroidal quantitative blood flow map (mL/mL/min) is calculated. Then along the length of the retina, either the peak BF values are taken from the retina and choroid (locations indicated with the red/blue lines) or the BF is integrated/summed over the retinal and choroidal depths (transparent regions). This provides BF values for the retina and choroid over the length of the retina, which are then averaged over the length for each animal.

60 · ΔM/[2α · T_1B_ · exp(-PLD/T_1B_) · exp(-TE/T_2B_) · λ_a_ · M_0_],

where λ_a_ is the volume of water per volume of arterial blood taken as 0.85 mL/g (40, 42, 43), T_1B_ is the T_1_ of arterial blood assumed to be 1.8 s at 7T, T_2B_ is the T_2_* of arterial blood assumed to be 35 ms at 7T, α is the label efficiency taken to be 0.75, ΔM is the difference between non-labeled and labeled images, and M_0_ is the signal of water as taken from the pre-retinal vitreous of each profile from the M_0_ image. The BF was then analyzed using two different approaches (**Figure 2**). First, the peak BF values (mL/mL/min) were taken from the retinal and choroidal vascular layers as previously reported (35, 44). Second, a partial volume correction was applied, as avascular regions bound the retina (the vitreous, sclera, and avascular outer retina), by integrating the BF across the retinal depth to give BF per retinal surface area (µL/mm^2^/min). This was done by rescaling the BF to units of µL blood/mm^3^ of tissue/min, and then summing BF across the depth of the retina or choroid layers and scaling by the resolution perpendicular to the retina (42 µm). For each approach, the BF was then averaged along ~1 mm lengths of the retina on both sides of the optic nerve head.

### 2.3 Simulations

**Figure 3:**
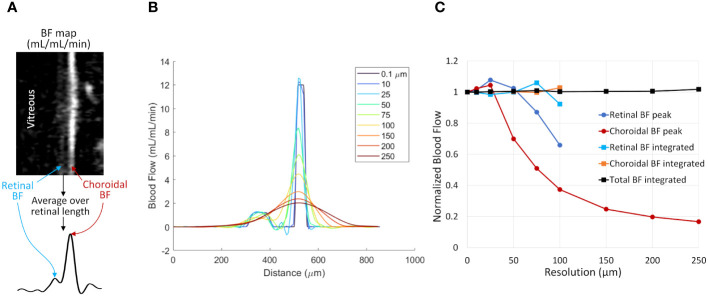
The effect of spatial resolution on retinal and choroidal blood flow measurements from simulated 1D profiles. **(A)** Depiction of 1D profiles across the retinal depth using *in vivo* data. A linearized BF map is shown with retinal and choroidal BF labeled. Averaging over the length of the retina provides a 1D profile across the depth. **(B)** Simulated 1D profile of retinal and choroidal BF using several spatial resolutions. The profile was simulated at 0.1 µm resolution and resampled at resolutions from 10 to 250 µm. **(C)** BF values were taken from the simulated profile by taking the peak value of each layer or by integrating across each layer. Data were normalized to the known simulated values from the original 0.1 µm data. Layer-specific values are given only for the resolutions where the retina and choroid could be resolved.

Simulations of BF data were performed to investigate the effects of partial volume on the accuracy of the two analysis methods for the relatively low spatial resolution of MRI. A 1D profile of the retinal and choroidal BF across the retinal depth was simulated, as such profiles are extracted and analyzed as in our previous studies methods (37, 38) and as depicted in **Figure 3A**. The simulation was performed to approximate the BF values and laminar thicknesses of the *in vivo* mouse retina. The BF profile was simulated at high spatial resolution (0.1 µm) and then resampled at multiple lower resolutions to cover the range comparable to MRI acquisitions (around 30-250 µm). The data were downsampled by taking the spatial Fourier transform of the profile and removing high spatial frequency components, to simulate MRI data acquisition which samples data in k-space. All downsampled data were then spatially interpolated to 7 µm, as in our analysis. The simulated data for multiple resolutions were then analyzed to take the peak values and integrated values.

### 2.4 Statistical analysis

Data are reported as mean ± standard deviation (SD). Statistically significant differences were considered with P *<* 0.05. To compare power using a single eye or both, group comparisons were made between DBA/2J mice and C57BL/6J mice using t-tests (Excel, Microsoft, Redmond, WA) for single eyes and linear mixed models with group and eye as fixed factors and subject as a random factor for both eyes (SAS 9.4, SAS Institute, Cary, NC). The significance of an association between BF and IOP was tested using linear mixed models with IOP as a covariate and subject as random factor for both eyes. The Pearson correlation coefficient and linear regression with 95% confidence intervals were calculated for each eye separately (R version 4.3.1). For power and effect size analyses, overall means and SDs for each group were taken by averaging the group mean and SD for each eye. To compare differences in statistical power between the two BF calculation approaches, effect sizes were calculated for between group comparisons (Cohen’s d) as the group mean differences divided by a pooled SD. Additionally, power analyses (G*Power 3.1.9.7, Heinrich-Heine-Universität Düsseldorf) (45) to estimate sample sizes needed for a power of 0.9 to detect group differences were performed for t-tests with a single eye and repeated ANOVA with two eyes using the group means, SDs, and between-eye Pearson correlation coefficients from the data.

## 3 Results

### 3.1 Dual coil phantom tests

**Table 1** shows the SNR and excitation power for the two-coil set for the left and right coils. There was no trend for reduced SNR or greater excitation power when using both coils, indicating negligible losses with the dual coil setup. Images were also viewed with scaling set to show the background noise, and there was no visible signal above the background noise level from the detuned coil. These results demonstrate the lack of interaction or losses due to using the dual coil setup.

**Table 1:** SNR and power with only a single coil used, both coils placed but only the imaging coil connected, and both coils placed and connected.

	SNR - RARE		SNR - FLASH		Excitation power (mW)	
	Left	Right	Left	Right	Left	Right
**Single coil**	141	161	170	182	39.8	32.4
**Imaging connected**	142	161	160	188	38.9	32.4
**Both connected**	161	163	190	183	32.4	33.1

### 3.2 Simulation of blood flow analysis methods

**Figure 3B** shows simulated retinal BF profiles, simulating acquisitions at different spatial resolutions. The simulated BF data were analyzed using the two analysis approaches (**Figure 3C**). At simulated resolutions lower than 100 µm the separate retinal and choroidal vascular layers could not be resolved, so only the choroidal BF peak and the total integrated BF (combined retinal and choroidal BF) are plotted. The peak BF values progressively declined as the sampled resolution approached the same size as the layer thickness, as this causes significant blurring of the layer. The integrated BF values, however, remained relatively stable, even as the resolution becomes relatively low.

### 3.3 Demographic and physiological parameters in mice

The mean ages were 11.3 ± 1.0 months for DBA/2J mice and 11.5 ± 0.8 months for C57BL/6J mice. The mean body weights were 34 ± 3.4 g for DBA/2J and 35.5 ± 2.4 g for C57BL/6. There were no significant differences between the age and weight of the mouse groups (P = 0.59 and 0.23, respectively). The DBA/2J mice had an intraocular pressure of 16.4 ± 2.6 (range, 13-21) mmHg in the left eye and 16.3 ± 1.9 (range, 14-20) mmHg in the right eye (**Table 2**). In comparison, the C57BL/6J mice had an overall lower intraocular pressure of 12.0 ± 1.0 (range, 11-14) mmHg in the left eye and 11.4 ± 1.1 (range, 10-14) in the right eye. DBA/2J compared to C57BL/6, had significantly higher IOP in both left and right eyes (P=7E-5 and 6E-7, respectively, by t-test). While previous studies report that optic nerve degeneration is asymmetric in DBA/2 mice (7, 46), IOP herein was significantly correlated between eyes in DBA/2J mice (Pearson correlation R=0.68, P=0.015), but not in C57BL/6J mice (R=0.48, P=0.11).

**Table 2:** Group-averaged (mean ± SD) intraocular pressure and P-values (t-test).

	IOP (mmHg)		
	DBA/2J	C57BL/6J	P
**Right eye**	16.3 ± 1.9	11.4 ± 1.1	6E-7
**Left eye**	16.4 ± 2.6	12.0 ± 1.0	7E-5

### 3.4 *In vivo* retinal and choroidal BF

**Figure 4:**
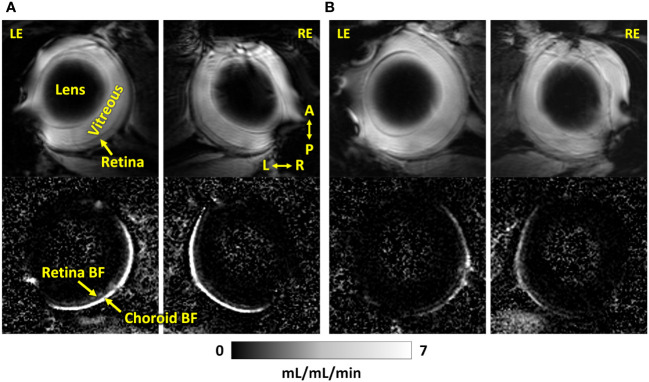
Representative MR images (top) and corresponding blood flow maps (bottom) using dual coils of both eyes of an **(A)** C57BL/6J mouse and a **(B)** DBA/2J mouse. LE, left eye; RE, right eye; A, anterior; P, posterior; L, left; R, right. The quantitative blood flow maps are scaled from 0 to 7 mL/mL/min.

**Figure 5:**
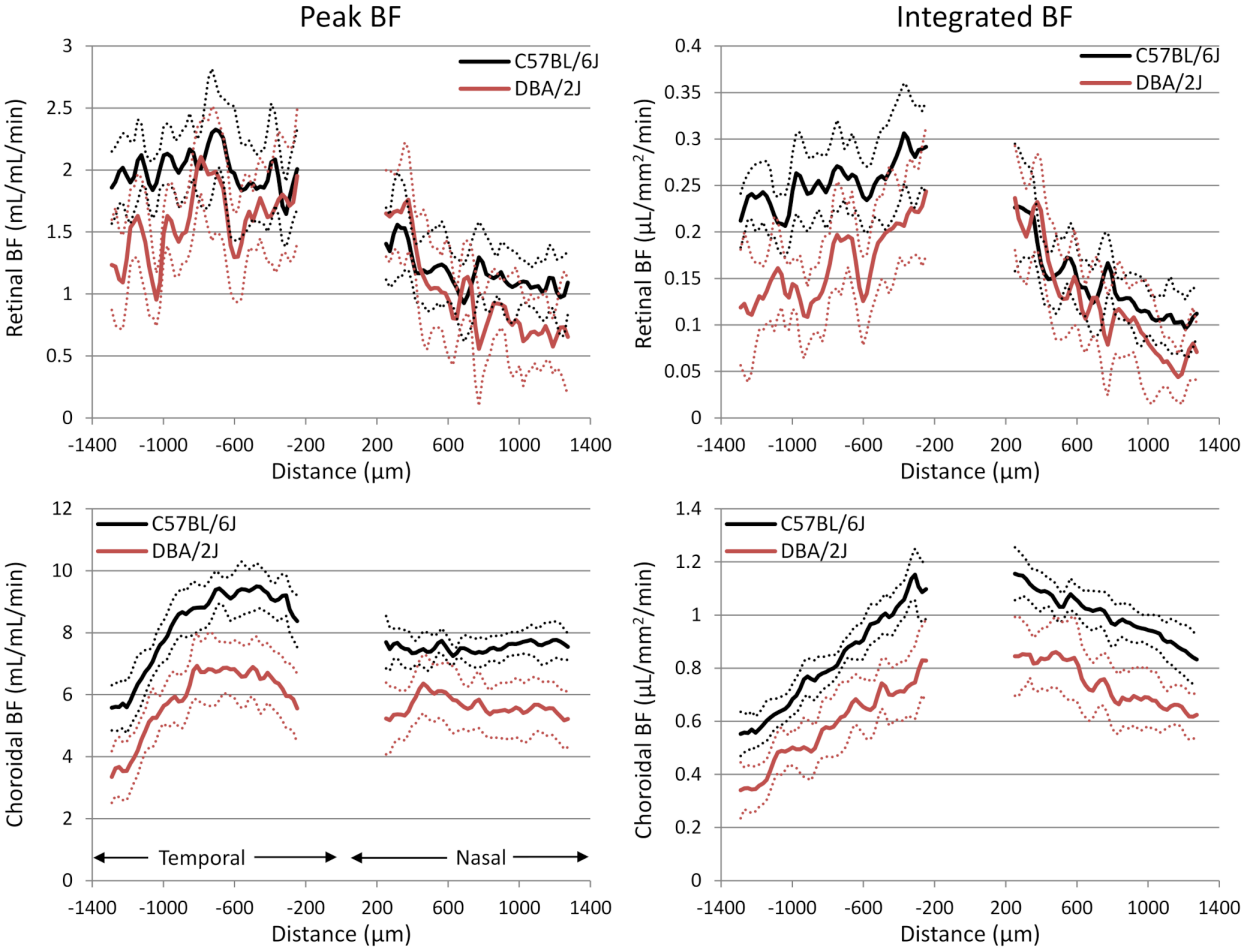
Group average blood flow plotted along the length of the retina from DBA/2J and C57BL/6J mice. Nasal and temporal sides are indicated. *Top row*: retinal BF, and *Bottom row:* choroidal BF. *Left column*: BF values taken from the peak value of each layer as flow per unit volume of tissue, and *Right column*: BF values from integrating over each layer as flow per unit surface area of tissue. Data from left and right eyes were averaged. Solid lines are means and dashed lines are 95% confidence intervals.

**Figure 6:**
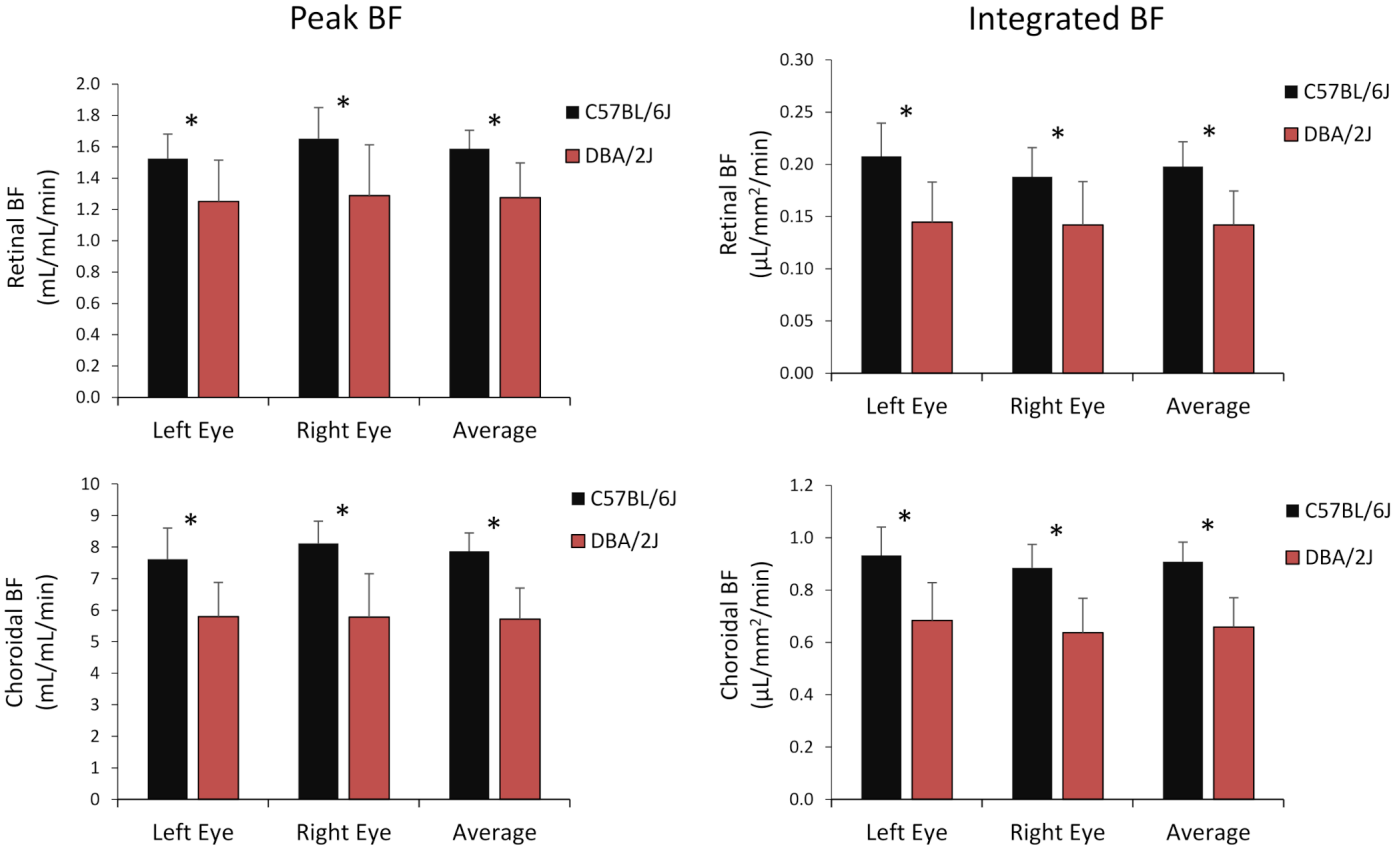
Group average blood flow from both eyes and the average of eyes of DBA/2J and C57BL/6J mice. *Top row*: retinal BF, and *Bottom row:* choroidal BF. *Left column*: BF values taken from the peak value of each layer as flow per unit volume of tissue, and *Right column*: BF values from integrating over each layer as flow per unit surface area of tissue. Mean ± standard deviation. * P<0.05 between C57BL/6J and DBA/2J by t-test.

**Figure 7:**
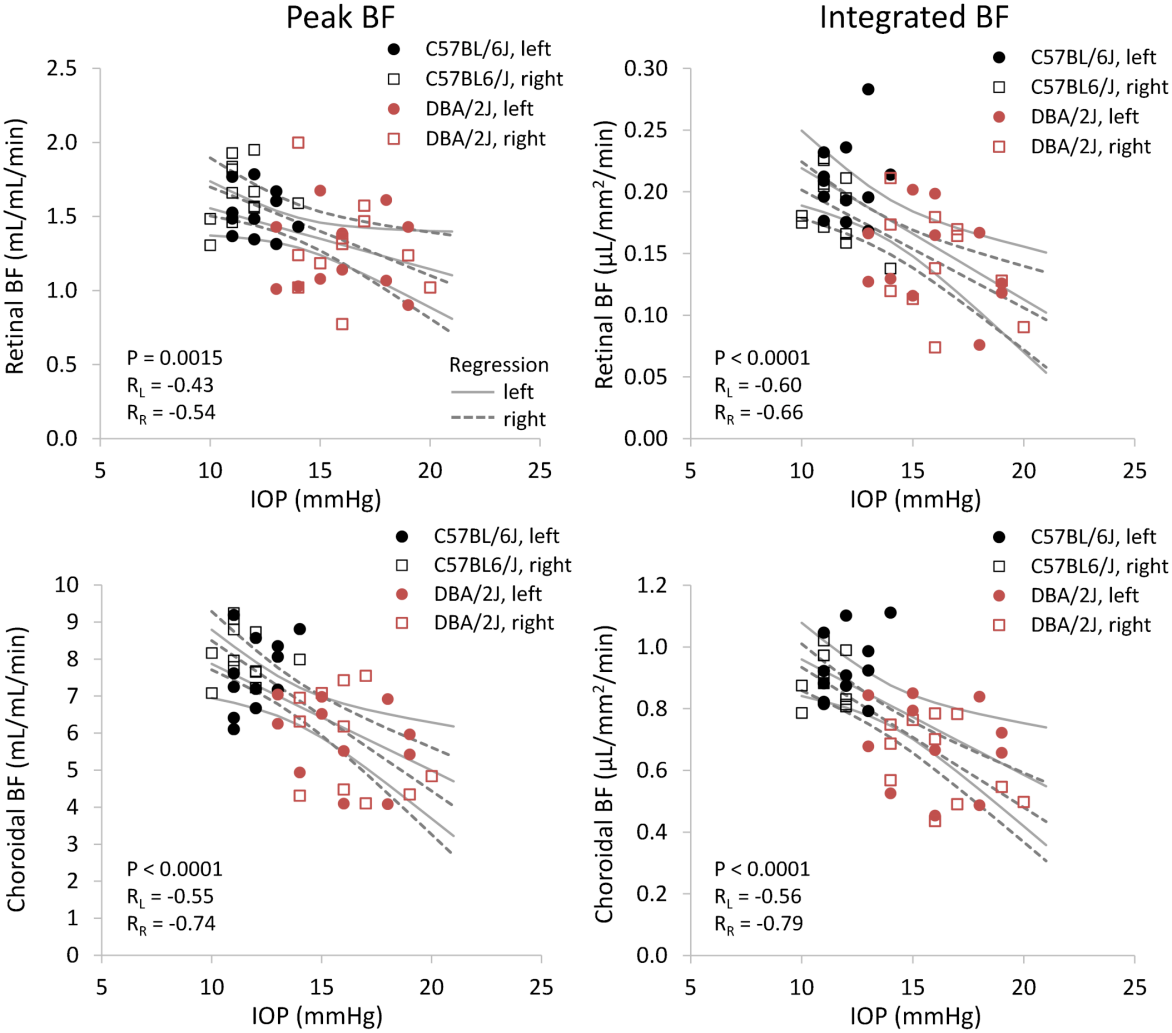
Scatter plots of blood flow and IOP from individual DBA/2J and C57BL/6J mice. *Top row*: retinal BF, and *Bottom row:* choroidal BF. *Left column*: BF values taken from the peak value of each layer as flow per unit volume of tissue, and *Right column*: BF values from integrating over each layer as flow per unit surface area of tissue. Linear regression lines and 95% confidence intervals are plotted with solid lines for left eyes and dashed lines for right eyes. The P-value including repeated measures for the effect of IOP on BF is given, and R_L_ and R_R_ indicate correlation coefficients for left and right eyes, respectively.

Representative BF images from C57BL/6J and DBA/2J mice are shown in **Figure 4**. BF along the length of the retina is shown for both analysis methods for C57BL/6J and DBA/2J mice in **Figure 5**. The BF was generally higher in the central retina and lower towards the periphery, although this trend was more apparent for the integrated calculation. Group averaged BF values and P-values for group comparisons using single eyes (t-tests) are given in **Table 3** and plotted in **Figure 6**. Retinal BF in both eyes was significantly lower in DBA/2J mice than in C57BL/6J mice using both analysis methods (P=0.0003 for peak and P=0.0001 for integrated, linear mixed model). Choroidal BF in both eyes was significantly lower in DBA/2J mice than in C57BL/6J mice using both analysis methods (P<0.0001 for peak and P<0.0001 for integrated, linear mixed model). The correlation coefficients for retinal BF were 0.40 and 0.59 for peak and integrated methods, and the correlation for choroidal BF were 0.54 and 0.65 for peak and integrated methods. The correlations using the integrated analysis method were stronger for both retinal and choroidal BF. **Figure 7** shows scatter plots of retinal and choroidal BF versus IOP for C57BL/6J and DBA/2J mice. Retinal and choroidal BF from both calculation methods were significantly associated with IOP (all P ≤ 0.0015), with lower BF with higher IOP. The correlation coefficients between IOP and BF for each eye are shown in **Figure 7** and were between -0.43 and -0.79 for all measures.

**Table 3:** Group-averaged (mean ± SD) retinal and choroidal blood flow and P-values (t-test).

		Peak BF (mL/mL/min)			Integrated BF (µL/mm²/min)		
		DBA/2J	C57BL/6J	P	DBA/2J	C57BL/6J	P
**Retina**	**RE**	1.29 ± 0.32	1.65 ± 0.20	0.0053	0.142 ± 0.042	0.188 ± 0.028	0.0063
	**LE**	1.25 ± 0.26	1.53 ± 0.16	0.0084	0.145 ± 0.038	0.208 ± 0.032	0.0004
**Choroid**	**RE**	5.78 ± 1.38	8.11 ± 0.71	0.0002	0.637 ± 0.131	0.885 ± 0.090	6E-5
	**LE**	5.79 ± 1.08	7.62 ± 0.98	0.0004	0.683 ± 0.144	0.932 ± 0.108	0.0002

RE, right eye; LE, left eye.

The effect sizes and sample size estimates for between group comparisons are shown in **Table 4**. Using data from both eyes gave a smaller estimated sample needed, as expected, albeit the gains for choroidal BF were minimal given its very large effect size in this data set. Comparing BF analysis approaches, the effect sizes were larger and estimated sample sizes smaller using the integrated approach. The practical gain for the choroid was again limited due to its large effect size, but using both eyes and the new integrated analysis approach reduced the estimated sample size for retinal BF by 38%.

**Table 4:** Effect size and sample size estimates for retinal and choroidal BF measurements.

	Retinal Blood Flow		Choroidal Blood Flow	
	Peak	Integrated	Peak	Integrated
**Cohen’s d**	1.33	1.56	1.99	2.08
**N – one eye**	13	10	7	6
**N – both eyes**	10	8	6	6

## 4 Discussion

The bilateral coil setup allowed the acquisition of data from both eyes simultaneously, while still maintaining the same time to acquire data. This could enhance statistical power by doubling sample size without requiring more mice, albeit with correlated measures between eyes. This method therefore has the potential to allow for fewer mice to be used in future studies, supporting the goal to minimize animal use while maximizing data quality. The proposed analysis approach to calculate BF per retinal surface area is expected to reduce partial volume effects and was also found to potentially improve statistical power. The DBA/2J mice in our study exhibit significantly elevated IOP at 11 months of age, as consistent with prior reports. DBA/2J mice herein had mean IOP values of more than 4 mmHg greater than age-matched C57BL/6J mice. Across both eyes, there was a consistent disparity in ocular blood circulation between the two groups. Retinal and choroidal BF was significantly decreased in the DBA/2J mice compared to the C57BL/6J mice, both per unit tissue volume and per unit tissue area. These results support prior BF studies in DBA/2J mice and show the potential of a bilateral coil setup and multiple methods of quantifying BF.

The results of reduced choroidal and retinal BF in DBA/2 mice are consistent with previous papers, which used only a single coil setup to image the left eye. These studies demonstrated a progressive age-related reduction in retinal and choroidal BF in DBA/2 mice (5, 26, 47). Compared to age-matched normotensive C57BL/6 mice, retinal and choroidal BF were significantly lower by 4 to 9 months (5, 26, 47). Retinal and choroidal BF were both significantly declined with age in DBA/2 mice, with lower BF in the mice at 6 and 9 months of age compared to 4 months of age (5). Alongside this pattern of declining BF, DBA/2 mice have an age-dependent elevation of IOP, typically found to be elevated by 9 months of age (8). Two studies using fluorescein angiography or histology reported that reduced retinal vessel density occurs in DBA/2J mice after 6 months and by 8-10 months of age and that retinal vascular leakage occurs by 10-11 months of age (48, 49). The MRI BF findings that retinal and choroidal BF are significantly reduced at 6 months of age compared to 4 months suggest that either BF deficits might precede vascular loss or else the reported vascular density methods were less sensitive. The hypoxic immunohistochemical marker hypoxia-inducible factor-1α (HIF-1α) was also reported to be elevated in DBA/2J mice by 6 months of age (49), consistent with the timing of reduced BF.

These BF and IOP findings are consistent with the literature on glaucoma, and additional MRI investigations on the changes of ocular BF in the face of varying IOP can further elucidate the pathophysiology ocular hypertension and the progression of glaucoma. A primate model of glaucoma reported a 40% reduction in total choroidal BF alongside large IOP elevations to 35-48 mmHg (10), compared to a 27% choroidal BF reduction found herein in mice. In an inducible rat model of ocular hypertension, retinal BF at five weeks after induction was reduced by about 50% alongside large IOP elevation to about 42 mmHg (50), compared to a 20-28% retinal BF reduction found herein in mice. Retinal vascular density was also slightly but significantly reduced by three days after induction and was substantially decreased by 60% at five weeks (50). The larger BF changes reported in induced models could likely be due to the much larger increases in IOP compared to the DBA/2J model. Impaired ocular hemodynamics have also been found in human glaucoma patients, as well as reduced vascular density of the retina, optic disc, and choroid (9, 11). The retinal blood vessels arise from the central retinal artery, while the choroidal vessels arise from the posterior ciliary arteries (PCA) (51). In the optic nerve head, the prelaminar and laminar regions are supplied primarily by PCAs and branches from the peripapillary choroid, while the retrolaminar region is supplied by both the PCAs and branches from the central retinal artery (52, 53). A study reported that reduced parapapillary choroidal vascular density from ocular coherence tomography angiography (OCTA) was associated with a marker of autonomic impairment in normal tension glaucoma (54). As only the choroidal vasculature has autonomic innervation and the choroid and much of the optic nerve head blood supply arise from the PCAs (19, 52), choroidal BF regulation and impairment may be relevant for glaucoma, albeit choroidal BF changes are not necessarily always reflective of the optic nerve head BF (52). Additionally, the hypoxic immunohistochemical marker HIF-1α in the retina and optic nerve has been noted in glaucoma models and patients (55, 56). This suggests that the decreased BF in the retina and optic nerve leads to tissue hypoxia in glaucoma. Additional MRI investigations on the changes of ocular BF during acute variations of IOP could further elucidate the pathophysiology of ocular hypertension and the progression of glaucoma.

Advantages of MRI are that it is non-invasive and provides volumetric BF in quantitative absolute units, which allows for longitudinal and cross-sectional comparisons. MRI has depth resolution and is not impacted by opacities blocking the view of the retina, such as cataracts. MRI measures the delivery of arterial BF to the capillary bed, which is quantified by the volume of blood delivered per unit time per quantity of tissue (57, 58), similar to gold-standard microsphere BF measurements. BF quantified in such units is a crucial hemodynamic parameter because it directly determines the delivery rate of oxygen and nutrients to the tissue, which is simply the BF value times a substrate’s arterial concentration (58). BF MRI with arterial spin labeling is well-validated in the brain with gold-standard PET and microspheres (59) and is reproducible in the brain and choroid (60, 61). Retinal BF from MRI has been validated with gold-standard microspheres in normal rats, with good agreement between methods (4). Choroidal BF also had good correlation between MRI and microspheres, although direct comparison of BF values was not possible due to limitations with quantification for the choroid in rodents by microspheres (4). Disadvantages of MRI are that the scans are relatively long (around 5-20 minutes) and relatively low resolution. Comparison between ocular BF methods is not trivial because they generally measure different hemodynamic parameters from different locations. Doppler methods measure blood velocity, rather than flow, from large branch retinal vessels. This can be converted to total retinal BF by combining with vessel diameter measurements but is only applicable to the retina and provides limited regional BF information (62, 63). Laser speckle contrast imaging (LSCI) provides relative indices of BF or velocity with high resolution and very rapid acquisition. Basal flow values are not absolute and can be influenced by characteristics of the optical pathway which complicates cross-sectional or long-term longitudinal comparisons (64, 65), but studies have reported differences such as in glaucoma (66). Several parameters related to changes over the cardiac cycle can be quantified. As LSCI does not have depth resolution, measurements will be some combination of retinal and choroidal flow. Numerous studies have utilized OCTA to image retinal and choroidal vessel density due to its very high-resolution with moderately rapid acquisition times (67–69). OCTA provides depth resolution of retinal and choroidal vasculatures and has good repeatability in the same setting (70). Disadvantages of OCTA include difficulty with quantitation, as the common vessel density index has large variability between studies, with values from about 25 to 90% (71–74). Quantitative validation of OCTA vessel density is also lacking, with one study using an ex vivo perfused eye preparation finding OCTA fails to detect some microvessels in the retina (75). Quantifying BF remains a challenge with OCTA, as standard OCTA signals primarily detect the presence of vessels and provide limited information on the BF speed. However, recent studies have explored methods to additionally provide a relative index of blood velocity in the vessels (76), which has been used to visualize regional variation in relative flow in disease (77). This approach has also been applied to track BF changes due to acute perturbations in rodents over a few hours (69), however its suitability for objective cross-sectional or longitudinal studies is unclear (69), which is needed for applications to glaucoma patients and the DBA/2J model used herein.

This paper provides a modified approach to measuring retinal and choroidal BF using MRI with bilateral coils as opposed to a single-coil approach. A few previous manganese-enhanced MRI studies used dual coils to image both eyes in rodents, but each eye was scanned sequentially using a single coil at a time due to the absence of detuning circuits, which would double the scan time (78–80). Some studies have also used a single large coil to image both eyes simultaneously with contrast-enhanced MRI, but with relatively low resolutions (>100 µm) that cannot resolve retinal/choroidal layers (81–84). These previous studies also have not incorporated a third transmit coil for blood labeling for ASL, as was used herein. The findings using the two coil method in glaucomatous mice are consistent to those using a single-coil method (5, 26, 47), as expected. Additionally, the two-coil method increased the statistical power of the BF measurements without requiring additional mice or scan time. This is consistent with reviews of statistical analysis for ocular data which recommend including data from both eyes if available as within-subjects measurements to improve power (85, 86).

We additionally tested a novel method of BF quantification by integrating BF measurements over the depth of each retinal layer, thereby calculating flow per surface area. This approach was expected to reduce partial volume effects inherent in thin retinal layers, which the simulation results supported. Peak BF values diminished with lower spatial resolution, while integrated values remained consistent. Since achieving sufficiently high resolution to completely avoid such partial volume effects with MRI is difficult, the integrated analysis approach could have benefits to improve accuracy of quantitative BF values in the retina. The statistical power was also increased using the integrated approach, which we speculate could be due to including a few additional voxels across the retinal depth instead of a single voxel for each peak. A previous study in humans used a similar approach for quantifying choroidal BF in µL/mm^2^/min but summed the BF in the anterior-posterior direction of a rectangular region, rather than profiles perpendicular to the curved retina as done herein, because the resolution on people was insufficient to resolve the curvature of the BF in the posterior eye (87). Additionally, only the choroidal BF can currently be resolved in human studies. In contrast, animal studies using microspheres and autoradiography have reported BF per retinal surface area from flat-mount retinal tissue. Choroidal BF in primates has been found to range from 7-10 µL/mm^2^/min in the central retina and 0.5-1 µL/mm^2^/min in the periphery, while rabbit choroidal BF fluctuates around 0.5-2.5 µL/mm^2^/min (27, 28). Estimating choroidal BF in rats from reported total choroidal BF values (µL/min) (3, 4), by converting to flow per surface area by assuming a 6 mm eye with the retina covering half the eye, gives around 1.3 to 3 µL/mm^2^/min. There appears to be a trend of lower choroidal BF per surface area in smaller eyes, which is consistent with our results in normal mice which are slightly lower than rats. This may be due to the much thinner choroid in smaller animals, for example around 300 µm in humans but only 25-45 µm in mice (88–90). Retinal BF in primates is 0.3 and 0.06 µL/mm^2^/min centrally and peripherally, respectively (27). Estimating retinal BF in rats from reported total retinal BF values (µL/min) gives around 0.16 to 0.34 µL/mm^2^/min (3, 4). These are similar to our mouse results, suggesting retinal BF per surface area is consistent across species with vascularized retinae.

The impact of anesthesia on hemodynamic measurements warrants careful consideration. Both retinal and choroidal circulations are affected by vasoconstrictive and vasodilatory anesthetics, such as isoflurane (35, 44). A study measuring anesthetic sensitivity to isoflurane in mouse strains showed there may be slight differences in anesthetic sensitivity to isoflurane between DBA/2 and C57BL/6 mice, but the overall differences seem minor (91). Blood pressure is similar in awake DBA/2J and C57BL/6J mice (92) and remains stable and similar between strains during one hour of isoflurane (5). The blood pressure and heart rate responses to hypercapnia are also similar in awake DBA/2J and C57BL/6J mice (93), while isoflurane reportedly has minimal effect on blood pH and pCO2 in C57BL/6J mice (94). Although anesthesia can influence physiological responses, the observed differences in ocular BF between DBA/2J and C57BL/6J mice are thus likely largely reflective of underlying disease mechanisms rather than the effects of anesthesia. However, measurements in a physiologically normative condition without anesthesia would better our understanding of the BF dynamics in glaucoma, although awake imaging of BF in the retina in small animals presents a challenge. Additionally, the time of day may affect ocular BF measurements. IOP is reported to have a circadian variation with higher values during the dark in several mouse strains, including DBA/2J and C57 mice (95, 96). Diurnal fluctuation of the ocular vasculature has also been reported in humans. Higher flow is reported in the optic nerve head at night with variation of around only 2% over the equivalent period in which measurements were made herein, and with the transitions between high/low flow occurring around the transitions between light and dark, periods in which we did not make measurements (97). The choroid flow is flat except for a brief peak in the early evening (97), a period in which we did not make measurements. In contrast, choroidal thickness and luminal area are reported to be largest in the early morning and smaller in the afternoon to night (98). As reported flow in humans is quite stable during the middle of the dark period, we do not expect major diurnal effects on BF measurements made over the time ranges used herein. For glaucoma, direct BF measurement of the optic nerve head at the laminar region would be of great interest. The capability to image BF of the optic nerve head with depth resolution (42 µm) in mice has been demonstrated (99). However, the very small size of the nerve and long scan times make this application sensitive to even minute eye motion. Other anesthetic and paralytic protocols may be needed (100) to allow routine application of BF MRI to the optic nerve head. One limitation with this analysis approach for layer-specific retinal and choroidal BF, is that with the current resolution there is still likely some partial volume between the tails of the retinal and choroidal BF layers. Based on the simulations, we expect this amount is relatively small and that both layers have similar amounts blurring into the other which would further reduce the effect on quantification with the integrated approach.

In conclusion, the dual eye coil method allows for blood flow MRI measurements to be obtained simultaneously from both eyes, increasing the amount of data acquired and improving statistical power without prolonging acquisition times. This would also provide the ability to study compensatory effects in unilateral pathology, such as following unilateral glaucoma surgery. MRI allows the measurement of absolute, quantitative BF per unit of tissue in the retina and choroid without depth limitation. This improved, two-coil MRI approach successfully replicated the results of previous studies that found a decrease in retinal and choroidal BF in DBA/2J mice compared to C57BL/6J mice. The pathological significance of ocular BF impairment in glaucoma remains uncertain. The improvements to the MRI ocular BF technique could be used to longitudinally investigate the role blood flow dysregulation has in glaucoma and retinal diseases such as diabetic retinopathy.

## Data availability statement

The original contributions presented in the study are included in the article/**Supplementary Material**. Further inquiries can be directed to the corresponding author.

## Ethics statement

The animal study was approved by Institutional Animal Care and Use Committee of Stony Brook University. The study was conducted in accordance with the local legislation and institutional requirements.

## Author contributions

ZJ: Conceptualization, Data curation, Investigation, Methodology, Writing – review & editing. DC: Data curation, Formal analysis, Visualization, Writing – original draft, Writing – review & editing. AG-C: Data curation, Formal analysis, Visualization, Writing – original draft, Writing – review & editing. DT: Formal analysis, Visualization, Writing – original draft, Writing – review & editing. RH: Conceptualization, Writing – review & editing. TD: Conceptualization, Funding acquisition, Writing – review & editing. EM: Conceptualization, Data curation, Formal analysis, Funding acquisition, Methodology, Project administration, Resources, Software, Supervision, Visualization, Writing – review & editing.

## Supplementary material

Supplementary Table 1 Intraocular pressure (IOP) for both eyes, body weight, and age for individual animals.

Supplementary Table 2 Retinal and choroidal blood flow (BF) peak values per volume of tissue (mL/mL/min) along the length of the retina for individual animals. Tabs include data for the distance from the optic nerve (µm) from which the BF data were taken, choroidal BF, and retinal BF for each eye. LE: left eye, RE: right eye. *indicates data which were discarded from statistical analysis as described in the methods.

Supplementary Table 3 Retinal and choroidal blood flow integrated values per surface area of tissue (µL/mm^2^/min) along the length of the retina for individual animals. Tabs include data for the distance from the optic nerve (µm) from which the BF data were taken, choroidal BF, and retinal BF for each eye. LE: left eye, RE: right eye. *indicates data which were discarded from statistical analysis as described in the methods.

Supplementary Table 4 Retinal and choroidal blood flow values averaged over about 2 mm lengths of the retina for individual animals. Peak values (mL/mL/min) and integrated values (µL/mm^2^/min) are given. *indicates data which were discarded from statistical analysis as described in the methods.
